# Protein Requirements in Critically Ill Older Adults

**DOI:** 10.3390/nu10030378

**Published:** 2018-03-20

**Authors:** Rachel R. Deer, Elena Volpi

**Affiliations:** 1 Division of Rehabilitation Sciences, The University of Texas Medical Branch, Galveston, TX 77555, USA; rrdeer@utmb.edu; 2 Sealy Center on Aging, The University of Texas Medical Branch, Galveston, TX 77555, USA; 3 Department of Internal Medicine, The University of Texas Medical Branch, Galveston, TX 77555, USA

**Keywords:** older adult, nutrition, hospitalization, protein

## Abstract

Critically ill elderly patients’ nutritional needs are not well understood and vary with the phase of illness and recovery. Patients’ nutritional needs should be assessed early in hospitalization and re-assessed throughout the stay with additional attention during the transitions from critical illness, to severe illness, to post-hospital rehabilitation. In this review, we summarize recent findings and highlight recommendations for protein supplementation in critically ill geriatric patients throughout the stages of recovery. Future research specifically focusing on protein dose, its relationship with caloric needs, and delivery modality must be conducted to provide more specific guidelines for clinical practice.

## 1. Introduction

The nutritional needs of critically ill geriatric patients (typically defined as individuals 65 years of age and older) are poorly understood. In addition, nutritional needs vary with the phase of illness and recovery—from critical illness, to severe illness, to post-hospital rehabilitation. In this review, we summarize recent findings and highlight recommendations for protein intake in critically ill geriatric patients throughout the stages of illness and recovery.

## 2. Protein Recommendations

Protein is the only macronutrient that does not have an inactive compound to serve as a reservoir in the way glycogen does for glucose or triglycerides do for fatty acids. Thus, dietary amino acids must be incorporated into functional proteins in order to be preserved from oxidation. Skeletal muscle contractile proteins are the largest protein reservoir, and respond anabolically to feeding. Muscle protein amino acids can be rapidly released and utilized by the entire organism during fasting or stress. Insufficient protein intake to satisfy daily requirements leads to negative protein balance and results in skeletal muscle atrophy, impaired muscle growth, and functional decline. Therefore, it is important that the proper amount of protein is consumed to prevent muscle wasting and maintain skeletal muscle mass and function. Food intake decreases dramatically during aging. Compared with younger adults, older adults on average eat slower, are less hungry and thirsty, consume smaller meals, snack less between meals, and have lower energy intakes [1]. Thus, it is important to educate older adults on foods that are healthy and calorically dense and to provide recommendations on ways to consume adequate doses of high-quality protein at each meal.

Muscle growth is dependent upon protein consumption and the resulting hyperaminoacidemia, which stimulates muscle protein synthesis (MPS) and, to a lesser extent, decreases muscle protein breakdown (MPB). When dietary protein consumption is insufficient to satisfy daily requirements, skeletal muscle atrophy occurs due to the negative protein balance (MPB is greater than MPS). This results in impaired muscle growth and functional decline. Utilization of dietary amino acids for MPS has been reported to be blunted or impaired in healthy older adults, compared to younger individuals. Metabolic studies have shown that this anabolic resistance can be overcome by higher levels of protein/amino acid intake [2,3]. Multiple studies have indicated that 25–30 g of a high quality protein is necessary to reach the threshold for maximal stimulation of MPS in older adults [4,5,6,7,8]. Thus, it is important to consume the quantity of protein that is necessary to optimally stimulate MPS in older adults in order to replenish the amino acids lost during fasting and during periods of catabolic stress such as hospitalization or critical illness. In light of these novel findings, the Recommended Dietary Allowance (RDA) for protein, which is based on nitrogen studies in younger and healthy individuals, is not very useful for guiding the dietary prescription of geriatric patients, particularly those who are critically ill. Indeed, results from the few nitrogen balance studies done in older adults are conflicting, with some supporting the current protein RDA [9] and others indicating that higher intakes are necessary to prevent nitrogen loss [10]. Additionally, two recent studies that employed the indicator amino acid technique found that the protein Estimated Average Requirement (EAR) for adults aged 65 and older is greater than the EAR reported by the Institute of Medicine [11,12]. Since the EAR is used to calculate the RDA, they concluded that both the EAR and RDA currently reported by the Institute of Medicine underestimate the real requirement and allowance for protein in older persons. Recent consensus reports have concluded that based on the available literature, the current protein recommendation for older adults is too low, and that older adults should consume more protein for optimal health [6,13,14,15]. 

It is important to note that protein requirements increase considerably with illness severity. Current clinical practice guidelines recommend giving patients with mild to moderate illness 0.8 to 1.2 g/kg protein per day, and to prescribe critically ill patients higher protein diets—1.2 to 1.5 g/kg protein per day [16]. 

## 3. Critical Illness and Recovery

During periods of high physical stress, like acute illness or hospitalization, the body undergoes an adaptive metabolic response to survive the acute illness. Patients experience multiple changes, including increased energy expenditure, hyperglycemia, loss of muscle mass and function, and eventually psychological and behavioral problems [17,18]. In addition, a major long-term consequence of stress metabolism is loss of muscle protein due to anabolic resistance and an increase in muscle protein breakdown [19]. Inactivity and systemic inflammation have been shown to exacerbate anabolic resistance, leading to muscle atrophy, increases in fat mass, and decrease in metabolic rate.

### 3.1. Critical Illness

During their stay in the Intensive Care Unit (ICU), patients are hypercatabolic due to a combination of the underlying disease, comorbidities, bed rest inactivity and poor nutritional intake. Diseases and conditions that land a geriatric patient in the ICU—such as sepsis, trauma, surgery, CHF, or respiratory failure—as well as contributing comorbidities (e.g., diabetes, COPD, CAD) have in common a high degree of inflammation with increased circulating cytokines, which activate protein catabolism and overall weight and lean mass loss [20,21]. Bed rest inactivity is another major contributor to lean mass loss. Several studies in healthy older adults have highlighted that even a short-term bed rest reduces appendicular lean mass, increases the age-related anabolic resistance to dietary amino acids [22] and increases expression of toll-like receptor 4 [23] and interleukine-6. In other words, bed rest inactivity predisposes the skeletal muscle tissue to produce an exaggerated response to noxious agents that accelerates net muscle catabolism. In the acute care setting, malnutrition is very common, occurring in 30–50% of hospitalized patients [24]. While some patients will be admitted to the ICU with a diagnosis of malnutrition, many will develop disease-induced acute malnutrition during their hospital stay [25]. 

Observational studies have reported contradictory findings, with large intakes of protein being associated with better [26,27] or worse [28] outcomes. In one study, large protein doses (1.5 g/kg/day) were found to be associated with the least negative total protein balance [29]. Much higher doses of daily protein intake of up to 2.5 g/kg/day have been proposed for ICU patients [30]. However, well-designed randomized controlled trials are needed to examine the association of better ICU outcomes with higher protein intakes. Care should be taken to examine study bias by illness severity, because sicker patients do not tend to tolerate feeding very well, leading to underfeeding and worse outcomes. 

Enteral nutrition—while potentially questionable under an ethical standpoint that goes beyond the scope of this review article—is preferred whenever possible to total parenteral nutrition, because it provides nutrients in a more physiologic manner. Enteral feeding supports the gut’s integrity, immune function and microbiome composition [31]. However, declining gut function and variable nutrient absorption rates may be problematic and can lead to gastrointestinal side effects and inadequate intake in critically ill patients [32]. As a result, enteral feeding may have to be supplemented or, in severe cases, substituted by parenteral nutrition, particularly in those patients with serious intestinal compromise (e.g., ileus, mesenteric infarct, malabsorption) or in those at high risk of aspiration. Nonetheless, it is important to underscore that parenteral nutrition increases the risk of hypercaloric feeding, hyperglycemia, and complications due to the indwelling central catheter. Recently, it has been proposed that rather than focusing on total caloric delivery in critically ill patients, clinicians should focus on the protein dose delivered either enterally and/or parenterally [33]. Currently, it is recommended to provide at least 1.5–1.8 g of protein/kg/day. If such a protein dose cannot be achieved with enteral nutrition, supplemental parenteral nutrition for provision of amino acids has been recommended [34]. However, these data have mostly been acquired in critically ill younger adults, and specific data in older critically ill patients are lacking. 

Another important question relates to which mode of feeding administration—continuous vs. intermittent boluses—is more effective in reducing catabolism and preventing muscle loss in older adults. In younger healthy individuals, intermittent feeding is more physiologic and can improve the whole body protein balance [35], while continuous feeding may lead to saturation of the protein anabolic response [36]. There are very few data in critically ill patients. A study reported improved whole body protein balance with intermittent amino acid infusion added to cyclic enteral feeding [37]. However, there are no specific data in critically ill geriatric patients.

Older patients also have higher prevalence of renal dysfunction and renal failure at admission to the ICU. This provides an additional challenge to providing adequate protein nutrition. In general, if the patient is undergoing dialysis protein needs are increased. Current recommendations can reach up to 2.5 g of protein/kg/day [38]. For those patients with moderately impaired renal function, the protein RDA of 0.8 g/kg/day is usually recommended in stable conditions, while during illness, it is recommended to increase protein intake to 1 g/kg/day to meet the higher demand [39]. Importantly, amino acid supplementation in critically ill patients with renal dysfunction did not worsen the duration of renal dysfunction [40]. 

Comprehensive guidelines for parenteral nutrition in critically ill patients and in geriatrics have been published by the European Society of Nutrition and Metabolism [41,42].

### 3.2. Severe Illness

As patients begin to recover from their critical illness and move from the ICU to non-ICU wards, they will also begin to transition from enteral and/or parenteral tube feeding to standard therapy with IV fluids and conversion to oral diet, as tolerated. An additional consideration should be made for the increased energy and protein needs as patients improve from being critically ill and bedridden to being ambulatory and able to participate in rehabilitation programs.

The prevalence and risk of malnutrition are high during hospitalization even in non-ICU units in geriatric patients. Many factors play a role in this increase, including high co-morbidity load, polypharmacy, depression, eating difficulties, functional limitations, decreased activity during hospitalization, cognitive impairment, and long periods of limited food intake due to procedures requiring nothing by mouth (nil per os—NPO—orders). In addition, older adults often need more time to eat than younger individuals, and may require assistance (e.g., cutting food, access to specialized instruments, help from someone to eat) [43]. 

Nutritional assessment should be performed on admission to the new ward and at frequent intervals throughout the patient’s hospital stay. While no gold standard exists, low body mass index and unintentional weight loss are often used as criteria in defining patients’ nutritional status. Numerous malnutrition screening tools have been validated for use in different populations or care settings (Subjective Global Assessment, Mini-Nutritional Assessment Short Form, Nutritional Risk Screening 2002, etc.) [44]. A multi-disciplinary approach to the plan of care should focus on the underlying causes of malnutrition, including co-morbid conditions, polypharmacy, depression, and social isolation/loneliness [45]. It is important to recognize patients who are at risk of malnutrition as early in the hospitalization as possible and a plan of care should be made to best benefit the individual patient. Research has shown that patients who are malnourished or at risk of malnutrition have greater average length of stay and higher costs per stay than patients with similar diagnoses who are not malnourished [46], as well as having increased mortality rates [47].

### 3.3. Post-Hospital Rehabilitation 

Another time that protein needs should be re-assessed is during post-hospital rehabilitation. As patients recover from hospitalization, care should be taken to encourage proper protein consumption after hospital discharge. Adequate nutrition during this high risk time frame has been shown to have positive benefits. 

Several studies have found that nutritional supplementation post-hospitalization can lower the incidence of falls, reduce inflammation, and increase handgrip strength [48,49,50]. In frail but otherwise-healthy older adults, an adequate (15 g) supplementation of high-quality protein at breakfast and lunch was shown to increase physical function. In addition, we recently reported that 30 days of protein supplementation (20 g twice daily) was feasible and increased physical function as measured by the short physical performance battery in older patients after an acute hospitalization [51,52]. The benefits of protein/amino acid supplementation on skeletal muscle health has been shown to be achievable during a 2–3 week time frame in clinical populations [53] or models of clinical settings [54]. Additional studies are needed to determine if supplementation is feasible during hospitalization and if post-discharge supplementation will provide functional benefits and reduce re-hospitalizations in more critically ill patients with longer lengths of hospital stays. 

## 4. Conclusions

In reviewing the existing literature, it has become evident that there are still many knowledge gaps that limit the establishment of specific protein recommendations for the various stages of critical illness in geriatric patients. Current clinical practice recommendations are to give patients with mild to moderate illness 0.8–1.2 g/kg protein per day, and to prescribe critically ill patients higher protein diets of 1.2–1.5 g/kg protein per day [16]. However, well-designed, randomized clinical trials are needed to clearly define the protein doses and amino acid composition (i.e., amount of leucine) that can provide the maximal health and functional benefits in critically ill geriatric patients. Specifically, it will be important to test increasing doses of protein in relation to the total caloric intake, as well as the modality by which protein is provided—enterally or parenterally—as well as the administration mode—intermittent vs. continuous vs. cyclic infusion. It will also be important to determine how much protein should be consumed by geriatric patients recovering from critical illness in order for them to regain physical and cognitive function and independence after the illness.
